# Patterns of Safety Incidents in a Neonatal Intensive Care Unit

**DOI:** 10.3389/fped.2021.664524

**Published:** 2021-06-10

**Authors:** Luise Brado, Susanne Tippmann, Daniel Schreiner, Jonas Scherer, Dorothea Plaschka, Eva Mildenberger, André Kidszun

**Affiliations:** ^1^Division of Neonatology, Department of Pediatrics, University Medical Center of the Johannes Gutenberg University Mainz, Mainz, Germany; ^2^Division of Neonatology, Department of Pediatrics, Inselspital, Bern University Hospital, University of Bern, Bern, Switzerland

**Keywords:** adverse event, neonatal care, safety incident, medical error, quality improvement

## Abstract

**Introduction:** Safety incidents preceding manifest adverse events are barely evaluated in neonatal intensive care units (NICUs). This study aimed at identifying frequency and patterns of safety incidents in our NICU.

**Methods:** A 6-month prospective clinical study was performed from May to October 2019 in a German 10-bed level III NICU. A voluntary, anonymous reporting system was introduced, and all neonatal team members were invited to complete paper-based questionnaires following each particular safety incident. Safety incidents were defined as safety-related events that were considered by the reporting team member as a “threat to the patient's well-being” which “should ideally not occur again.”

**Results:** In total, 198 safety incidents were analyzed. With 179 patients admitted, the incident/admission ratio was 1.11. Medication errors (*n* = 94, 47%) and equipment problems (*n* = 54, 27%) were most commonly reported. Diagnostic errors (*n* = 19, 10%), communication problems (*n* = 12, 6%), errors in documentation (*n* = 9, 5%) and hygiene problems (*n* = 10, 5%) were less frequent. Most safety incidents were noticed after 4–12 (*n* = 52, 26%) and 12–24 h (*n* = 47, 24%), respectively. Actual harm to the patient was reported in 17 cases (9%) but no life-threatening or serious events occurred. Of all safety incidents, 184 (93%) were considered to have been preventable or likely preventable. Suggestions for improvement were made in 132 cases (67%). Most often, implementation of computer-assisted tools and processes were proposed.

**Conclusion:** This study confirms the occurrence of various safety incidents in the NICU. To improve quality of care, a graduated approach tailored to the specific problems appears to be prudent.

## Introduction

Since publication of “To err is human” (1), health care professionals have steadily increased efforts to identify medical errors, to understand their causes, to find systematic mistakes and to improve patient safety. A number of strategies, like confidential reporting systems, direct observation, incident reporting systems (including voluntary reports), use of triggers, and chart reviews were established (2). Studies mainly focused on the detection of medication errors, one of the leading causes of adverse events (AE) (2–7). According to Sharek et al. a drug or non-drug related AE can be defined as “an injury, large or small, caused by the use (or non-use) of a drug, test, or medical treatment” (4). Caeymaex et al. defined patient safety as avoidance and prevention of patient injuries or AEs resulting from the process of health care delivery (8). Terminology of AEs differs not only across studies but also across organizations (3, 4, 6, 9). Gipson et al. have shown that the US Food and Drug Administration (FDA) and World Health Organization (WHO) use different terminologies for AEs (10). What most definitions have in common is that an AE is an untoward medical occurrence or an unfavorable and unintended sign, symptom, or illness.

The neonatal population is at increased risk for adverse drug events and a broad range of other adverse events (3, 4, 9, 11). The rate of adverse drug events is higher in the neonatal intensive care unit (NICU) than in adult intensive care medicine (5). Adverse drug events (including nutrition) (3, 9), errors of treatment (3), patient misidentification (3) and incidents in diagnostic procedures (9) are adverse events most frequently reported in the NICU. Evidence on frequency and patterns of safety incidents, which may precede adverse events, is sparse. In particular, there is little evidence from voluntary, interprofessional reports by NICU team members. However, safety incident reporting could be an important strategy for quality improvement (11). This study aimed at identifying patterns of safety incidents. Safety incidents are understood as safety-related events that were considered as a “threat to the patient's well-being” which “should ideally not occur again.”

## Methods

A 6-month prospective clinical study was performed from May to October 2019 in a German 10-bed level III NICU. The 10-bed NICU is a tertiary care facility, which admits an average of 70 very low birth weight infants per year. Of these, around 50% have a birth weight of <1,000 g and around 7% a birth weight <500 g, respectively. The NICU also cares for more mature infants with respiratory or circulatory support, surgical patients, and maintains a transport service for sick newborns from surrounding maternity hospitals. The majority of infants are inborn and are admitted immediately after birth, but the NICU also accepts infants transferred from other hospitals, readmissions from the special care unit and infants following discharge home. As part of a quality improvement (QI) initiative, a voluntary, anonymous reporting system was introduced. All neonatal team members (nurses and physicians) were asked to complete paper-based questionnaires following each safety incident which was noticed.

During a 3-month introduction period, all team members were repeatedly informed about the introduction of a new voluntary, anonymous and blame-free reporting system. All identifying patient data, such as age and gender were excluded from the report. We used an incident reporting form which was adapted on the basis of a previous publication by Suresh et al. (3). This reporting form was divided into eight sections:

1. Category of safety incident (medication error (including transfusion, nutrition, fluid substitution and electrolyte management); diagnostic error (delayed, wrong, or missed diagnosis); communication and patient misidentification; equipment problem and patient monitoring; errors in documentation; hygiene problems).
2. Type of safety incident (falsely or not prescribed, falsely or not administered/prepared, not or too late recognized, non-/dysfunction, misleading or missed communication).
3. Narrative section to describe the safety incident.
4. Estimation of preventability (preventable, likely preventable, likely non-preventable, non-preventable).
5. Time span until safety incident was recognized (near-miss, <4, 4–12, 12–24 h, 1-3 days, more than 3 days, unknown).
6. Severity of safety incident (incident would not have harmed, incident could have harmed but did not reach the patient, incident reached the patient but did not harm, incident potentially harmed, incident actually harmed [extended clinical monitoring or treatment] or seriously harmed [life-threatening] the patient).
7. Narrative section to describe actual damage to the patient.
8. Proposals of changes to prevent any such safety incident in the future.

All team members were encouraged to complete the reporting form and deposit it in a letterbox whenever recognizing a safety incident. The reports were collected monthly and finally analyzed following the 6-month study period.

## Results

During the study period, 179 patients were admitted to the NICU, and a total of 204 safety incidents were reported. Six reports were excluded from the analysis due to duplicate reports (*n* = 2) and reports not related to medical errors (*n* = 4). One hundred ninety-eight safety incidents were analyzed in 6 different categories (Table 1). The safety incident/admission ratio was 1.11.

**Table 1:** Distribution of safety incidents.

**Category**	**No. of incidents (*n* = 198)**
Medication errors (including transfusion, nutrition, fluid, and electrolyte management)	94 (47%)
Diagnostic errors	19 (10%)
Communication and patient misidentification	12 (6%)
Equipment problems and patient monitoring	54 (27%)
Errors in documentation	9 (5%)
Hygiene problems	10 (5%)

Medication errors were most frequently reported and essentially consisted of erroneous prescriptions in 55/198 (28%) cases and incorrect administrations in 36/198 cases (18%).

No serious or life-threatening events were reported. However, patients were considered to have been actually harmed in 17 cases (9%). Safety incidents which potentially harmed the patient were reported in 71 cases (36%). Details are given in Table 2.

**Table 2:** Distribution of severity of safety incidents.

**Category**	**Number (N) of**	**No harm^a^**	**Potential harm**	**Actual harm^b^**
	**safety incidents**	**N (%)**	**N (%)**	**N (%)**
Medication errors	94	52 (55.3)	37 (39.4)	5 (5.3)
Diagnostic errors	19	8 (42.1)	8 (42.1)	3 (15.8)
Communication and patient misidentification	12	9 (75.0)	1 (8.3)	2 (16.7)
Equipment problems and patient monitoring	54	24 (44.4)	23 (42.6)	7 (13.0)
Errors in documentation	9	7 (77.8)	2 (22.2)	0
Hygiene problems	10	10 (100.0)	0	0
Total (all safety incidents)	198	110 (55.6)	71 (35.9)	17 (8.6)

a *subsumes incidents that would not have harmed, incidents that could have harmed but did not reach the patient, and incidents that reached the patient but did not harm*;

b *no serious (life-threatening) incidents were reported*.

Of all safety incidents, 184/198 (93%) were considered to be preventable or likely preventable (Table 3).

**Table 3:** Estimation of preventability of safety incidents.

**Safety incident considered as …**	**No. of safety incidents (%)**
Preventable	**170 (86%)**
Medication errors	84 (42%)
Diagnostic errors	18 (9%)
Communication and patient misidentification	10 (5%)
Equipment problems and patient monitoring	41 (21%)
Errors in documentation	7 (4%)
Hygiene problems	10 (5%)
Likely preventable	**14 (7%)**
Medication errors	7 (3.5%)
Diagnostic errors	1 (0.5%)
Communication and patient misidentification	2 (1%)
Equipment problems and patient monitoring	3 (1.5%)
Errors in documentation	2 (1%)
Likely non-preventable	**9 (4.5%)**
Medication errors	4 (2%)
Equipment dysfunction/patient monitoring	5 (2.5%)
Non-preventable	**5 (2.5%)**
Equipment dysfunction/patient monitoring	5 (2.5%)

*The bold values represent the number of safety incidents in each of the four categories*.

Most safety incidents were noticed after 4–12 (*n* = 51, 26%) and 12–24 h (*n* = 48, 24%), respectively. Thirty-three (17%) safety incidents were detected in <4 h or were near-misses (*n* = 32, 16%). Twelve (6%) safety incidents were detected within 1–3 days, 11 (5%) safety incidents after three or more days. The period until the safety incident was recognized was unknown in 11 (5%) cases.

Medication errors were most often recognized as near-miss (*n* = 31, 16%). Equipment errors (*n* = 19, 10%) were most often detected in <4 h following the event. For a minority of 6 percent of all safety incidents (*n* = 11), it took more than 3 days until they were recognized.

Suggestions for improvement were made in 135/198 (68%) cases. Three suggestions were excluded as they were not related to avoidance of safety incidents. Therefore, 132/198 (67%) suggestions were analyzed. Most often, implementation of computer-assisted tools and processes were proposed (33/132, 25%). Other suggestions were implementing new standard operating procedures (SOPs) or consequently follow existing SOPs (32/132, 24%), and frequent training of clinical workflows and procedures (24/198, 12%). Furthermore, raising attention and awareness on the occurrence of medical errors (25/132, 19%) and improving equipment functionality (17/132, 13%) were considered to be helpful to reduce safety incidents.

## Discussion

This study confirms the occurrence of various safety incidents in the NICU. Medication errors, errors in fluid management and nutrition, equipment problems and erroneous processing of diagnostic results were frequently reported. Around nine percent of all safety incidents resulted into actual harm to the patient and most incidents were considered to be preventable.

We report an event/admission rate of 0.54 for medication errors and a rate of 1.11 for all safety incidents. Our data support the findings of Snijders et al. who also used a voluntary reporting system that detected an event/admission rate of 0.53 for adverse drug events and 1.26 for all safety incidents (9). Other reports show similar frequencies of adverse events. Palmero et al. (2) report an event/admission rate of 2.34, and, using a trigger tool method, Sharek et al. report an event/admission rate of 0.74 (4). In Sharek's study, 65% percent of all adverse events were deemed preventable, which appears less than what we have observed (4). However, preventability is not an objective criterion and reflects individual and collective estimates, which may vary significantly across institutions.

Our results are content to previous studies. Safety incidents are multifaceted and adverse drug events were most often recorded (3, 4, 6, 9). Similar to our results, Suresh et al. report, that the most frequent event categories were adverse drug events (47%), errors of treatment (14%), patient misidentification (11%), other system failure (9%), error or delay in diagnosis (7%), and error in the conduct of an operation, procedure, or test (4%) (3). They furthermore report that failure to follow the policy or protocol (47%), inattention (27%), error in charting or documentation (13%), distraction (12%), inexperience (10%), labeling error (10%), and poor teamwork (9%) were supposed to be the most frequent contributory factors (3). It appears as Suresh et al. detected more communication problems (22%) when compared to our study (12%). Although not verifiable, this difference is most likely explained by different NICU sizes. Our NICU is rather small, with less personnel involved in clinical care, when compared to the participating NICUs in Suresh's study.

Like Suresh et al., Snijders et al. also showed that medication errors occurred most frequently (27%), followed by laboratory (10%) and enteral nutrition errors (8%). A number of studies focused on adverse drug events, in particular (2–7). According to Meyer-Massetti et al. incident reports do identify the fewest medication errors but are effective in capturing the incidence of severe drug-related issues (12). However, methodological differences and ambiguous terminology make comparisons across studies difficult (2–4, 9, 10).

Suresh et al. reported that 27% of all medical errors resulted in harm to the patient which was considered to be a major problem (3), whereas Snijders et al. stated that the majority of incidents had no actual consequences at the time of reporting, but many reported incidents were thought to be potentially harmful (9). They also reported that severe harm occurred in 7/4,846 (0.1%), and moderate harm during 63/4,846 (1.3%) safety incidents (9). We have observed in our study that actual harm occurred in almost 1 out of 10 safety incidents, but no serious harm. Although medication errors constituted the largest group of safety incidents in our study, safety incidents due to equipment failures, diagnostic and communication errors appeared to be especially problematic, since these incidents were more often considered to have actually harmed the patient. Safety incidents are remarkably heterogeneous with respect to their manifestation and also their consequences for patient safety. Safety incidents appear to vary in severity depending on the category in which they occur.

Particular strengths of our study are its interprofessional approach and the detailed characterization of safety incidents. Our study has, nevertheless, pilot character and several limitations. The true incidence of safety incidents remains unknown, as the voluntary reporting system is selective and incomplete. Therefore, our results cannot be easily transferred to other cohorts. The 6-month study period might be considered insufficient to detect the overall frequency of safety incidents, especially in a rather small NICU. Furthermore, anonymous reporting hampers an analysis of how errors arise. On the other hand, anonymous reporting supports the team members to feel free to report without fear of punishment and blame (13, 14). When kept simple, regular reporting appears feasible in the complex daily routine in the NICU (15).

However, our results are hardly generalizable and must be interpreted with caution since the definition of harm was broad and individual assessments on what constituted harm to the patient will have differed. Other limitations of our study include its rather small sample size and short duration. The completely anonymous nature of the survey does not allow any conclusions to be drawn as to which professional group reported which errors.

Further studies will need to prove that safety incident reporting results in the implementation of effective preventive strategies. Such strategies might include team resource management trainings, introduction of double-check approaches and electronic patient data management systems. Implementation of preventive strategies and evaluation of their effectiveness is a time-consuming and difficult process that has already been investigated in several studies (11, 16, 17). In one of those studies, van der Starre et al. found that only around one third of specific recommendations derived from medical errors, were actually implemented (11). They also identified specific barriers to their implementation, e.g., no fixed assignments of responsibility, unspecific recommendations, or reliance on external factors. Failure to adequately respond to safety incidents can be very discouraging and further complicate the implementation process (11). Future studies will need to address the relationship between safety incidents with and without effects on the patient and especially, whether and how a reduction of all safety incidents will affect the occurrence of serious, harmful events. A graduated approach, tailored to specific problems might be necessary during QI initiatives.

In summary, safety incidents were frequently reported in our NICU. Further research is necessary to characterize critical safety incidents and to determine the relationship between safety incidents and manifest adverse events. Detection of safety incidents appears to be an essential prerequisite to improve quality of care and ensure patient safety. Since most safety incidents were deemed preventable, implementation of QI appears to be prudent and promising. Strategies should be tailored specifically and be primarily focussed on medication errors, equipment functionality and processing of diagnostic results.

## Data Availability Statement

The raw data supporting the conclusions of this article will be made available by the authors on reasonable request.

## Author Contributions

LB contributed to conception, design of the study and the questionnaire, contributed to data collection, analyzed data, and wrote the first draft of the manuscript. ST, DS, JS, and DP contributed to data collection, edited the manuscript, and approved the submitted version. EM contributed to design of the study and to data collection, edited the questionnaire, and the manuscript. AK conceived the idea of the study, contributed to conception and design of the study, edited the questionnaire, contributed to data collection and analysis, and edited the manuscript. All authors contributed to the article and approved the submitted version.

## Conflict of Interest

The authors declare that the research was conducted in the absence of any commercial or financial relationships that could be construed as a potential conflict of interest.
